# Modelling transmission and control of *Toxoplasma gondii* in New Zealand farmland

**DOI:** 10.1098/rsos.241282

**Published:** 2025-02-05

**Authors:** Rachelle N. Binny, Graham J. Hickling, Alex James, Chris N. Niebuhr

**Affiliations:** ^1^ Manaaki Whenua – Landcare Research, Lincoln, New Zealand; ^2^ School of Mathematics and Statistics, University of Canterbury, Christchurch, New Zealand

**Keywords:** toxoplasmosis, predator control, feral cat, rodents, epidemiological model, differential equations

## Abstract

*Toxoplasma gondii* is one of the world’s most prevalent parasites and has significant impacts on the health of humans, domestic animals and wildlife. In New Zealand’s rural environments, *T. gondii* creates economic losses for the farming industry and threatens vulnerable native avifauna and marine mammals. Predator control of rodents and feral cats has potential to reduce or even eliminate transmission of *T. gondii* on farms; however, the efficacy of such management is uncertain. We apply a mathematical model of *T. gondii* transmission dynamics in feral cat and rodent populations in New Zealand farmland and simulate varying intensities of predator control to predict changes in *T. gondii* prevalence and environmental contamination levels over time. The model predicts that predator control is relatively ineffective for reducing transmission in areas with high environmental contamination rates. However, assuming low rates of environmental contamination, local elimination of *T. gondii* could be achievable, for example, by control that sustains large reductions of 88%, 69% and 59% in feral cat, house mouse and ship rat populations, respectively, over 56 weeks. Predator control is, therefore, a potentially viable approach for managing *T. gondii* in some rural environments, but only if high levels of population control are sustained.

## 1. Introduction

The intracellular protozoan *Toxoplasma gondii*, the causative agent of toxoplasmosis, is a highly transmissible parasite of worldwide distribution. Infection is common in many warm-blooded vertebrates, including humans, domestic animals and wildlife [1]. In humans, *T. gondii* infections are widespread, with prevalence varying by location, and certain strains present a public health risk via foodborne transmission [2]. Domestic pigs, sheep and goats are at particularly high risk of infection by *T. gondii*, where it is a common cause of abortion [3,4]. *T. gondii* also poses a threat to wildlife populations, including the Hawaiian monk seal (*Neomonachus schauinslandi*) and sea otter (*Enhydra lutris*) in the USA, and the Hector’s and Māui dolphins (*Cephalorhynchus hectori hectori* and *C. h. maui*, respectively) in New Zealand [5]. Clinical toxoplasmosis has also been reported in terrestrial New Zealand wildlife, including kākā (*Nestor meridionalis*), red-crowned parakeet/kākāriki (*Cyanoramphus novaezelandiae*), kererū (*Hemiphaga novaeseelandiae*) and kiwi (*Apteryx* spp.) [6].

The life cycle of *T. gondii* is complex, consisting of both definitive and intermediate hosts. Felids are the only definitive host, of which only *Felis catus* (both domestic and feral) is present in the New Zealand landscape. In cat definitive hosts, the parasite reproduces sexually to produce oocysts that are shed into the environment in faeces 3−21 days post-infection, with peak oocyst excretion occurring between 6 and 8 days [5]. Once in the environment, oocysts sporulate and become infectious within 1−5 days or longer, depending on environmental conditions [7]. Infectious oocysts can survive in the environment for over a year and may diffuse through soil or be transported in waterways, which can also lead to contamination of freshwater and marine environments [8]. Intermediate hosts (warm-blooded animals), in which the parasite undergoes asexual reproduction, are infected by ingesting oocysts, typically from contaminated soil, water and plants. Once ingested, the oocysts develop into mobile tachyzoites, then into quiescent tissue cysts called bradyzoites, often in the neural and muscle tissues. In some species, including rodents, congenital transmission may also occur from mother to offspring [9]. Cat definitive hosts, while capable of ingesting oocysts from the contaminated environment, also acquire infection by consuming raw or undercooked meat containing tissue cysts. However, while cats may be infected by either mode, oocysts have lower infectivity to cats than to intermediate hosts and ingestion of tissue cysts in infected prey is probably the more efficient transmission pathway [10]. Following primary infection, usually as post-weaned kittens, cats typically gain immunity to *T. gondii*, and while they may experience waning immunity later in life, it is uncommon that further oocyst shedding occurs in adult cats. There are therefore three main transmission pathways by which *T. gondii* is considered to spread: infection by oocysts from contaminated environments, congenital (vertical) transmission, and carnivorism [11].

In New Zealand farmland, non-native small mammal species, primarily mice (*Mus musculus*), ship rats (*Rattus rattus*), Norway rats (*R. norvegicus*) and lagomorphs (mostly rabbits, *Oryctolagus cuniculus*), as well as small bird species, are important prey of feral cats [12]. A camera trapping study in Hawke’s Bay farmland found that cat site occupancy was positively correlated with rodent abundance, with over 90% of identified *Rattus* species being ship rats [13]. Rodents are likely to be important intermediate hosts for *T. gondii* in farmland, but the prevalence of *T. gondii* in New Zealand’s cat and rodent populations is largely unknown. In a review of *T. gondii* infections in wild rodent populations worldwide between 2009 and 2020, Dubey *et al*. [14] reported prevalence ranging from 0 to 58.6% for ship rats (mean 18.2%, 22 studies), and from 0 to 36.6% for house mice (mean 14.8%, 10 studies), with prevalence in grasslands generally much higher than in forest [15]. Preliminary estimates from a pilot study looking at *T. gondii* infection levels in ship and Norway rats in New Zealand showed 10% (6/60) and 15% (3/20) of individuals were infected, respectively (C.N.N. 2024, personal communication).

Management of toxoplasmosis on New Zealand farms is currently achieved through vaccination of sheep; however, there are currently no commercially available vaccines to reduce *T. gondii* infection in humans or wildlife species, or to reduce oocyst shedding in cats [16]. Predator control of non-native mammals, typically by trapping and toxic baiting, is also frequently conducted on New Zealand farmland to reduce crop damage, prevent disease spread (e.g. spread of bovine tuberculosis by brushtail possum (*Trichosurus vulpecula*) [17]), or protect native flora and fauna [18]. It has been suggested that rodent and cat control could contribute to reducing or eliminating transmission of *T. gondii* [6,19,20]. However the likely efficacy of such management is uncertain. A large-scale predator control programme targeting feral cats and mustelids (and rodents as by-catch) in Hawke’s Bay did not measurably reduce seroprevalence of *T. gondii* in one-year-old ewes over three years; it is unclear whether higher removal rates or longer durations of sustained control could have had an impact [21]. Given the difficulty and expense of such field studies, we adopted a modelling approach to investigate the potential for predator control as a tactic to reduce *T. gondii* transmission under New Zealand conditions.

Several models of *T. gondii* transmission have been developed. Turner *et al*. [20] extended a differential equation susceptible–infected–recovered (SIR) model by Lélu *et al*. [22], describing the dynamics of susceptible, infected and recovered populations of cats and mice, and the level of oocyst contamination in the environment. The model included the effects of varying virulence of different strains, vertical transmission in rodents, infection-induced behavioural changes in mice, vaccination of cats and harvest control of mice. These models have been adapted to consider the effectiveness of cat vaccination for reducing oocyst-originated *T. gondii* infections in humans [23] or reducing prevalence in endangered native carnivores [24]. Rasambainarivo *et al*. [24] found that very high cat vaccination rates or large reductions in rodent populations were required to significantly reduce the risk of *T. gondii* infection in wild carnivores in Madagascar. Spatially explicit models have also been applied to predict effects of landscape structure on spatial distribution of *T. gondii* in rural environments [25].

An important measure of the transmission potential of a disease is the basic reproduction number, $R_{0}$ , i.e. the expected number of secondary cases generated by a single infectious individual in a completely susceptible population. Infection will continue to spread if $R_{0}$ > 1 and will go extinct if $R_{0}$ < 1. By calculating $R_{0}$ , Lélu *et al*. [22] and Turner *et al*. [20] investigated the situations under which infection could persist within each of three transmission pathways (environmental infection of cats; vertical transmission in mice; and the predator–prey cycle), as well as the contributions of these pathways to transmission along gradients of predation rates and cat population sizes.

In this paper, we model *T. gondii* transmission and predator–prey dynamics in cat and rodent populations, using a compartment model based on those of Lélu *et al*. [22] and Turner *et al*. [20]. We parameterize the model for application in the context of New Zealand farmland and extend it to include transmission dynamics in ship rat populations, and population control of cats and rodents by trapping and/or toxic baiting. We then use the model to predict the change in prevalence of *T. gondii* in rodent and cat populations over time, and to explore the likely effectiveness of rodent and cat control for on-farm toxoplasmosis management. We also investigate model parameters with the greatest uncertainty to assess their relative contributions to the disease dynamics and thereby prioritize variables for empirical measurement.

## 2. Methods

**Figure 1.**
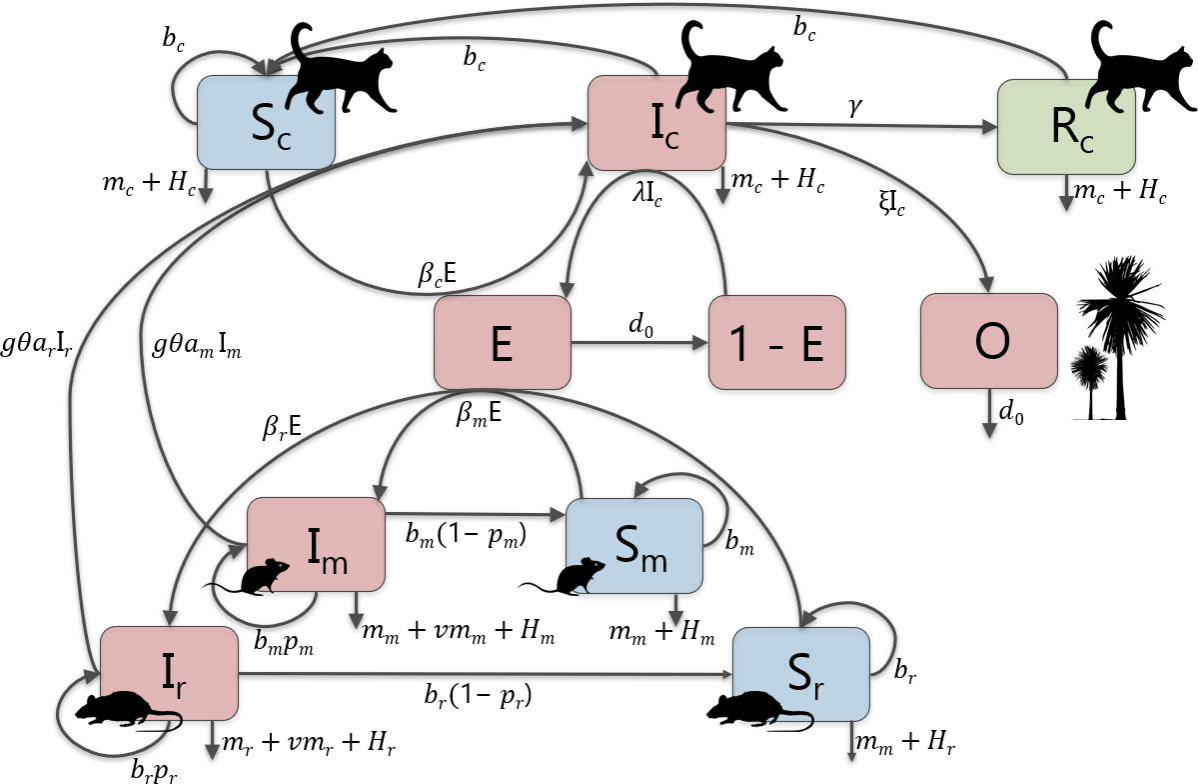
Schematic of compartmental SIR model of *T*. *gondii* transmission for susceptible (*S*), infected (*I*) and recovered (*R*) individuals in feral cat (*c*), house mouse (*m*) and ship rat (*r*) populations, and the oocyst-contaminated environment (proportion of defecation sites that are contaminated, *E*, and the number of oocysts, *O*). Full explanation of parameters in text.

We extend the SIR models of Lélu *et al*. [22] and Turner *et al.* [20] describing parasite transmission in feral cat and house mouse populations for the life cycle of *T. gondii*, to include ship rats as a second intermediate host, and modelling cat population control instead of cat vaccination. The model represents the rates of change in the contaminated environment, in susceptible and infected individuals of the three host species, and in recovered individuals in the cat population. We incorporate population growth, predation of cats on rodents, environmental transmission, vertical transmission in rodents and control (see figure 1). The model assumes homogeneous mixing of individuals and that there is no immigration/emigration of any species.

### 2.1. Feral cat population

We model the cat population in three compartments representing cats that are susceptible to infection $S_{c}$ , infected cats $I_{c}$ , and those that have recovered from infection (i.e. are no longer infectious after acquiring lifelong immunity) $R_{c}$ . The total size of the cat population is denoted $N_{c}$ = $S_{c}+I_{c}+R_{c}$ . The number of individuals in each compartment changes over time according to the following ordinary differential equations (ODEs):

**Figure:**
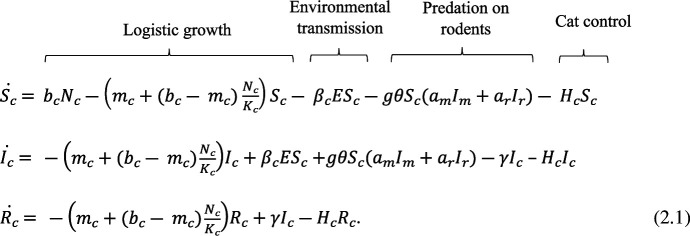


Population growth is logistic with an intrinsic growth rate *r*_*c*_ = *b*_*c*_ – *m*_*c*_, comprised of intrinsic (*per capita*) rates of birth *b*_*c*_ and mortality *m*_*c*_. Density-dependent effects driven by resource limitation reduce the growth of the population as its size approaches a carrying capacity *K*_*c*_.

There are two pathways of *T. gondii* transmission in cats: transmission via predation of infected prey and transmission from the contaminated environment (e.g. soil, water). Cats acquire infection by consuming tissue cysts in infected prey with probability *g* = 1, representative of the type II lineage of *T. gondii*, which is particularly prevalent worldwide so likely has a high chance of infecting cats if they ingest tissue cysts. Cats can also become infected by direct contact with the oocyst-contaminated environment with transmission rate $\beta_{c}$ , but this pathway is assumed to contribute less to overall transmission in cats compared to the predation pathway, due to the known low infectivity of oocysts to cats. Infected cats recover at a rate γ . We incorporate sustained control (e.g. by trapping) of the cat population by assuming trapping removes a constant proportion $H_{c}$ of the cat population from the $S_{c},I_{c}$ and $R_{c}$ compartments, with a total $H_{c}N_{c}$ individuals captured per week.

### 2.2. Environmental contamination

We model the areas where infected cats defecate and excrete oocysts into the environment using two compartments: the proportion of defecation sites that are contaminated *E* and the proportion that are uncontaminated (1 – *E*). The rate of change $\dot{E}$ in environmental contamination is increased by infected cats shedding oocysts into uncontaminated sites at a rate λ and decreased by decay of oocysts in contaminated sites at a rate $d_{0}$ . This model formulation $\overset{˙}{E}=\lambda I_{c}(1-E)-d_{0}E$ , proposed by Lélu *et al.* [22], implies that oocyst contamination is clustered in localized defecation sites. Contamination can be considered a binary variable, i.e. defecation sites are either ‘contaminated’ or ‘uncontaminated’ and contact with a contaminated site will transmit infection with a constant probability of ingestion and infection. This is convenient as it allows the extent of environmental contamination to be considered as a proportion so *E* has a value between 0 and 1. As Bonačić Marinović *et al*. [23] discuss, an alternative approach by Turner *et al*. [20] was to remove the ${-\lambda I}_{c}E$ term from the rate of change equation. This instead implies homogeneous spread of oocyst contamination across the landscape and defines *E* as a continuous quantity proportional to the total number of oocysts (i.e. *E* can be a value > 1). This latter approach may be suitable under an assumption that transmission is more likely in heavily contaminated areas compared to those with low-level contamination. However, we consider the former approach more appropriate in the New Zealand farmland context; i.e. cats likely excrete oocysts in their faeces at discrete sites [12,26]. So, regardless of the number of oocysts, we assume a constant probability of infection given contact with a contaminated site. While transmission depends on *E*, managers may also wish to track the total oocyst load in the environment, for example to understand infection risk for other species due to ingesting a certain dose of oocysts (oocyst dose–response relationship). Following Bonačić Marinović *et al*. [23], the equation $\dot{O}={\xi I}_{c}-d_{0}O$ describes the change in total number of oocysts O in the environment over time, where ξ is the oocyst shedding rate of an infected cat.

### 2.3. Rodent populations

We model the ship rat population following the same dynamics as the house mouse population described by Turner *et al*. [20], but with different rates of birth, mortality and predation by cats. For brevity, we describe only the transmission dynamics for the house mouse population here; equations for the ship rat dynamics are identical to those for mice except subscripts *m* are replaced with *r*.

We assume that rodents suffer lifelong infections, and do not recover. The mouse population is modelled in two compartments for susceptible $S_{m}$ and infected $I_{m}$ mice with total population size $N_{m}$ = $S_{m}+I_{m}$ . Mouse populations undergo logistic growth in the absence of disease and control, with intrinsic growth rate *r*_*m*_ = *b*_*m*_ – *m*_*m*_, comprised of intrinsic (*per capita*) rates of birth *b*_*m*_ and mortality *m*_*m*_. Infection with *T. gondii* contributes an additional component *vm*_*m*_ to the mortality rate for mice in the infected compartment $I_{m}$ .

We model two pathways of transmission to rodents. Mice can be infected through ingestion of oocysts in the contaminated environment with transmission rate $\beta_{m}$ . They may also acquire infection congenitally via vertical transmission from infected parents to offspring [9,27–29]. Given a parent mouse is infected, we assume a constant probability $p_{m}$ of the parasite being transmitted to offspring.

We assume a type I functional response of predation (i.e. prey consumption rate of cats is a linear function of prey density) with cats predating on house mice with rate $a_{m}$ and ship rats with rate $a_{r}$ . When mouse and ship rat population sizes are at their equilibriums, $K_{m}^{*}$ and $K_{r}^{*}$ , respectively, each cat is expected to consume $a_{m}K_{m}^{*}$ mice and ${a_{r}K}_{r}^{*}$ ship rats per week in the absence of disease. There is evidence that *T. gondii* can modify the behaviour of rodent hosts such that they lose their aversion to predators [30], have higher activity levels [31,32], reduced locomotor performance [33] and lower neophobia [32]. We model these infection-induced behavioural changes as a change to the predation rate by a factor θ . Here, we assume θ > 1, indicating that infected rodents are at increased risk of predation. However, it is also possible to model no behavioural change or behavioural changes that increase aversion to predators by setting θ=1 or 0 ≤ θ < 1, respectively. When there is endemic infection in the rodent populations, the number of prey consumed by one cat therefore increases to a maximum $a_{m}$ ( $S_{m}^{*}+$ $\theta I_{m}^{*}$ ) mice and $a_{r}$ ( $S_{r}^{*}+$ $\theta I_{r}^{*}$ ) rats per week at equilibrium, where $S_{m}^{*}$ , $S_{r}^{*}$ and $I_{m}^{*}$ , $I_{r}^{*}$ denote the number of susceptible and infected mice and rats at equilibrium, respectively.

We incorporate sustained control (e.g. by trapping and/or application of toxic bait) of rodents by assuming that a constant proportion $H_{m}$ of the susceptible *$S_{m}$* and infected $I_{m}$ mouse populations are killed by control operations per week, and similarly for rats.

### 2.4. Transmission dynamics

The full dynamics of *T. gondii* transmission in cats, house mice and ship rats is represented by the following system of ODEs, describing rates of change in *S*, *I*, *R*, *E* and *O* compartments:


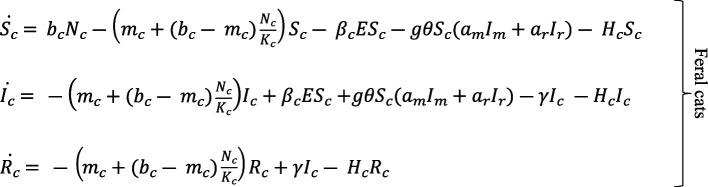



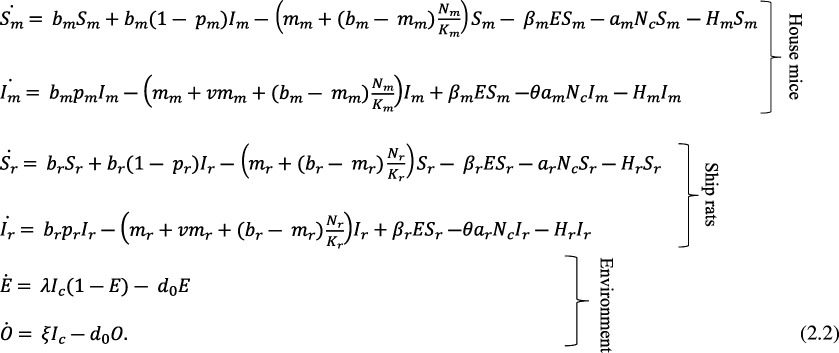


All species undergo logistic growth, with rodent populations facing additional mortality due to predation by cats, pest control and virulence of *T. gondii*. We assume the cat population dynamics do not depend (via predation) on prey population size because cats can consume prey species other than rodents. The dynamics of the sizes of the cat and rodent populations are


$$\overset{˙}{N_{c}}=\overset{˙}{S_{c}}+\overset{˙}{I_{c}}+\overset{˙}{R_{c}}=r_{c}N_{c}\left(1-\frac{N_{c}}{K_{c}}\right)-H_{c}N_{c},$$

$$\overset{˙}{N_{m}}=\overset{˙}{S_{m}}+\overset{˙}{I_{m}}=r_{m}N_{m}\left(1-\frac{N_{m}}{K_{m}}\right)-a_{m}(S_{m}+\theta I_{m})N_{c}-vm_{m}I_{m}-H_{m}N_{m},$$

(2.3) $$\overset{˙}{N_{r}}=\overset{˙}{S_{r}}+\overset{˙}{I_{r}}=r_{r}N_{r}\left(1-\frac{N_{r}}{K_{r}}\right)-a_{r}(S_{r}+\theta I_{r})N_{c}-vm_{r}I_{r}-H_{r}N_{r}.$$

Since cat survival is unaffected by infection, the size of the cat population at equilibrium is independent of the transmission dynamics, with


(2.4) $$\begin{array}{l} N_{c}\to \left\{ \begin{array}{l} 0\;\;\;\;\;\;if\;r_{c}-H_{c}<0\\ K_{c}^{*}\;\;if\;r_{c}-H_{c}>0 \end{array}\right. \end{array}\;\;\;as\;\;t\to \infty ,$$

where $K_{c}^{*}=(r_{c}-H_{c})\frac{K_{c}}{r_{c}}$ . In the absence of cat control, $N_{c}\to K_{c}$ as t→∞ . We begin by assuming several parameter constraints that prevent the extinction of any species:


$$r_{c}-H_{c}\;>\;0,$$

$$r_{m}-a_{m}\theta K_{c}^{*}-vm_{m}-H_{m}>0$$

and


(2.5) $$r_{r}-a_{r}\theta K_{c}^{*}-vm_{r}-H_{r}>0.$$

However, in order to assess how reducing rodent and/or cat populations to near-zero or zero abundance affects *T. gondii* transmission, we also consider scenarios where control rates $H_{m},\;H_{r}$ and $H_{c}$ are increased to values that violate these constraints.

Equilibrium rodent population sizes depend on the prevalence of infection, via the infection-induced behavioural changes affecting predation risk and the increased mortality from infection. In the absence of disease, the size of the house mouse population tends to an equilibrium $K_{m}^{*}$ = $(r_{m}-a_{m}K_{c}^{*}-H_{m})\frac{K_{m}}{r_{m}}$ . When disease is endemic, the equilibrium mouse population size has an upper limit at the disease-free equilibrium and a lower limit at $(r_{m}-a_{m}\theta K_{c}^{*}-vm_{m}-H_{m})\frac{K_{m}}{r_{m}}$ . Expressions for the lower and upper limits of the equilibrium for ship rat population size are identical to those for house mice but with the corresponding ship rat parameters.

### 2.5. Parameters for New Zealand farmland

We calibrate the model for *T. gondii* transmission in rural farmland in New Zealand’s Hawke’s Bay region, for a hypothetical site of area 10 km^2^ comprising a mosaic of pasture, scrub and native forest. A list of the parameter values used for Results is provided in table 1 and we conduct sensitivity analyses for certain parameters in electronic supplementary material, C. We used reported estimates of reproductive rates (a variety of measures), litter sizes and lifespans to calculate birth [37,43–47] and mortality rates [22,38,39,43,44,48,49]. Carrying capacities were chosen to lie within the ranges of population densities reported for New Zealand farmland sites [37,40,50,51]. Rates of cat predation on rodents range widely depending on prey availability and environment. Lélu *et al*. [22] assumed that predation rates between 0 and 21 prey cat^−1^ yr^−1^ (the value reported for domestic cats in an urban study [52]) represent urban environments while higher values correspond to suburban and rural sites. However, they and Turner *et al*. [20] restricted their analyses to rates below 52 prey cat^−1^ yr^−1^. Informed by a Hawke’s Bay farmland study of feral cat diet [12], we apply considerably higher predation rates that correspond to 255 rodent prey cat^−1^ yr^−1^, while noting that field studies have reported rates as high as 1892 prey cat^−1^ yr^−1^ (i.e. 72% of prey consumed out of 7.2 prey killed cat^−1^ d^−1^) in rural environments [53]. Under such high predation, the predator–prey cycle has a stronger influence on sustaining *T. gondii* infection than the environmental transmission cycle [20,22].

**Table 1.** Model parameters. To calibrate the model for New Zealand farmland, we assume a hypothetical site area of 10 km^2^. Time units are in weeks.

parameter	value	source
*environment*		
rate at which cats contaminate the environment	λ=0. 1/16 (low environmental contamination); λ= 1/16 (high environmental contamination) cat^−1^ week^−1^	[22,34]
environment decontamination rate	$d_{0}=7/100$ week^−1^	[22,35]
oocyst shedding rate of infected cats	$\xi =2\times 10^{8}$ cat^−1^ week^−1^	[23,36]
*feral cat*		
*per capita* birth rate	$b_{c}=5/52$ cat^−1^ week^−1^	[37]
*per capita* mortality rate	$m_{c}=0.6/52$ cat^−1^ week^−1^	[22,38,39]
carrying capacity	$K_{c}=40$ cats (i.e. 4 cats km^−2^)	[37,40]
predation rates on mice and rats	$a_{m}=3.709\times 10^{-4}$ ; $a_{r}$ = 3.235 × 10^−4^ cat^−1^ week^−1^	[12]
transmission rate from contaminated environment to cats	$\beta_{c}$ = 0.54/52 infections per infectious contact with contaminated site per week	[22,34,41]
probability of infection through consuming infected prey	*g =* 1	[20]
recovery rate of infected cats	γ = 0.5 week^−1^	[22,42]
control rate	$H_{c}=0.021$ (low), 0.042 (moderate), 0.063 (high), 0.080 (extreme) cat^−1^ week^−1^	see text in Methods
*rodent prey*		
*per capita* birth rate	$b_{m}=15/52$ mouse^−1^ week^−1^; $b_{r}=6/52$ rat^−1^ week^−1^	[43–47]
*per capita* mortality rate	$m_{m}=1.33/52$ mouse^−1^ week^−1^; $m_{r}=1/52$ rat^−1^ week^−1^	[43,44,48,49]
carrying capacity	$K_{m}=$ 10 000; $K_{r}=$ 5000	[50,51]
increase in mortality from infection	*v =* 0.3	[20]
transmission rate from contaminated environment to rodents	$\beta_{m}$ = 2.3/52, $\beta_{r}=1.05/52$ (low environmental contamination); $\beta_{m}$ = 0.9/52, $\beta_{r}=0.45/52$ (high environmental contamination) infections per infectious contact with contaminated site per week	see text in Methods
probability of congenital infection in rodents	$p_{m}=p_{r}=$ 0.75	[9,20]
infection-induced behavioural change affecting predation risk	θ=1.5	[20,30,33]
control rate	$H_{m}=0.0615$ (low), 0.1229 (moderate), 0.1844 (high), 0.2340 (extreme) mouse^−1^ week^−1^; $H_{r}=$ 0.020 (low), 0.041 (moderate), 0.061 (high), 0.077 (extreme) rat^−1^ week^−1^	see text in Methods

Environmental contamination rates are unknown for rural areas so we consider scenarios of ‘low’ and ‘high’ environmental contamination using two different values of λ . Decontamination rate was based on estimates of *T. gondii* survival in soil. While oocysts are nearly 100 times more infectious to mice than cats [5], the rates at which rodents and cats come into contact with oocyst-contaminated sites are also unknown. We assume the same value for $\beta_{c}$ as Lélu *et al*. [22] and choose values of $\beta_{m}$ and $\beta_{r}$ such that rodent infection prevalences at equilibrium are approximately 15%, i.e. close to the mean prevalence reported for ship rats and house mice [14]. Note that values of $\beta_{m}$ and $\beta_{r}$ differ between the two environmental contamination scenarios. In sensitivity analyses (see electronic supplementary material, C), we also vary $\beta_{m}$ and $\beta_{r}$ to compare ‘high’ and ‘low’ environmental transmission, corresponding to rodent prevalences close to 40% and 5%, respectively. Cat recovery rate was calculated assuming a 14 day infectious period. Evidence of vertical transmission in *Rattus* species is highly limited so we assume the same probability of vertical transmission for both rodent species, $p_{m}=p_{r}=0.75$ , based on the rate reported for house mice by Hide *et al*. [9] and as applied in the Turner *et al*. [20] model.

Control rates for cats and rodents vary depending on management objectives and methods. To explore the effectiveness of sustained cat and rodent control for managing *T. gondii* spread, we consider control rates between 0 and a maximum $H_{c}=$ 0.080, $H_{m}=$ 0.234 and $H_{r}=0.077$ , where these maximums are the control rates that achieve an approximately 95% reduction in the equilibrium abundance of cats, house mice and ship rats when populations are under single-species control. Note, however, that the size of rodent population reduction from control depends on cat abundance (via predation rate); for cat control rate $H_{c}=$ 0.080, higher values of $H_{m}=$ 0.250 and $H_{r}=0.091$ are required to achieve an approximately 95% reduction in rodent populations. In particular, we compare ‘no control’ ( $H_{c}=H_{m}=H_{r}=0$ ) with four categories of ‘low’, ‘moderate’, ‘high’, and ‘extreme’ control intensities that achieve approximately 25, 50, 75 and 95% reductions, respectively, in the equilibrium abundance of each species under single-species control (parameter values in table 1). A detailed description of parameter value calculations and sources is provided in electronic supplementary material, A.

### 2.6. Numerical analysis

A detailed investigation of the influence of the various transmission pathways on the life cycle of *T. gondii* is provided in Turner *et al*. [20] and Lélu *et al*. [22]; we do not replicate those results here but rather consider the full life cycle with a focus on assessing whether rodent and cat control could be a viable and effective approach to reducing *T. gondii* transmission in a rural farmland environment.

The nine compartments in equation (2.2) can be partitioned into five infected compartments, $\dot{I_{c},}\dot{I_{m}},\dot{I_{r}}$ , $\dot{E}$ and $\dot{O}$ , and four non-infected compartments, ${\dot{S}}_{c},{\dot{R}}_{c},{\dot{S}}_{m}$ and ${\dot{S}}_{r}$ . We refer to the equations for the infected compartments as the ‘infected subsystem’. The full system of ODEs has a disease-free equilibrium (DFE), $x_{0}$ , i.e. a steady state where there are no infected individuals $I_{c}=R_{c}=I_{m}=I_{r}=E=O=0$ , and an endemic equilibrium (EE) where infection persists. We do not derive analytical expressions for the equilibrium state of the full system, though it is straightforward to show that $\;E^{*}=\;\lambda I_{c}^{*}/(\lambda I_{c}^{*}+d_{0})$ and $O^{*}=\xi I_{c}^{*}/d_{0}$ , i.e. environmental contamination is an increasing function of the number of infected cats $I_{c}^{*}$ at equilibrium.

The DFE is given by


$$x_{0}={\left(S_{c}^{*},\;I_{c}^{*},\;R_{c}^{*},\;S_{m}^{*},\;I_{m}^{*},\;S_{r}^{*},\;I_{r}^{*},\;E^{*},O^{*}\right)}^{\prime }={\left(K_{c}^{*},\;0,\;0,\;K_{m}^{*},\;0,\;K_{r}^{*},0,\;0,0\right)}^{\prime }.$$

Furthermore, the DFE population sizes for cats, mice and rodents are: $N_{c}^{*}=S_{c}^{*}=K_{c}^{*}=(r_{c}-H_{c})\frac{K_{c}}{r_{c}}$ , $N_{m}^{*}=S_{m}^{*}=K_{m}^{*}=(r_{m}-a_{m}K_{c}^{*}-H_{m})\frac{K_{m}}{r_{m}}$ , and $N_{r}^{*}=S_{r}^{*}=K_{r}^{*}=(r_{r}-a_{r}K_{c}^{*}-H_{r})\frac{K_{r}}{r_{r}}$ .

We considered the potential for *T. gondii* to spread through a rural farmland environment by calculating the basic reproduction number $R_{0}$ for the model (see electronic supplementary material, B). $R_{0}$ is a threshold for local stability of the DFE such that the DFE is stable when $R_{0}\leq 1$ and infections die out, while if $R_{0}>1$ the DFE is unstable and the system instead tends to the EE. Following the methods of Diekmann *et al*. [54,55] and van den Driessche & Watmough [56], we calculate $R_{0}$ by first linearizing the infected subsystem around the DFE, which reflects that $R_{0}$ describes the potential for initial transmission of the parasite introduced into a fully susceptible population. We compute a next-generation matrix (NGM), which relates the number of newly infected individuals in each infected compartment in consecutive generations; $R_{0}$ is the dominant eigenvalue (spectral radius) of this matrix.

We also consider a scenario where rodent prey is completely eradicated by control (or naturally absent) and assess whether *T. gondii* can persist solely through transmission between cats and the environment. Note that the model does not consider non-rodent prey species, so this represents a theoretical scenario where there is no transmission of *T. gondii* between cats and rodents, nor any other infected prey species, e.g. lagomorphs or birds. Following rodent eradication, $S_{m}=I_{m}=S_{r}=I_{r}=0$ , the full system of equations—equation (2.2)—reduces to the subsystem ( $\dot{S_{c}}$ , $\dot{I_{c}}$ , $\dot{R_{c}}$ , $\overset{˙}{E}{)}^{\prime }$ which has a basic reproduction number (see electronic supplementary material, B, for calculations)


(2.6) $$R_{0}=\sqrt{\frac{\beta_{c}K_{c}^{*}\lambda }{d_{0}\left(b_{c}+\gamma \right)}}.$$

Finally, to consider the effectiveness of cat and rodent control for managing the spread of *T. gondii*, we compared the basic reproduction number $R_{0}$ , proportion of defecation sites that are contaminated $E^{*}$ , total number of oocysts in the environment $O^{*}$ , seroprevalence $R_{c}^{*}/N_{c}^{*}$ and infection prevalence $I_{c}^{*}/N_{c}^{*}$ of cats, and infection prevalence of house mice $I_{m}^{*}/N_{m}^{*}$ and ship rats $I_{r}^{*}/N_{r}^{*}$ at equilibrium for a range of control rates. The system of equation (2.2) was solved numerically using ‘scipy.integrate.odeint’ in Python’s SciPy package and the abundance of individuals in each compartment assessed at equilibrium. We used the same initial conditions for all scenarios, choosing initial abundances lower than carrying capacity for each species and setting the proportion of environment that was contaminated to 0.1. When estimating what period of sustained control is required to eliminate *T. gondii*, we used the equilibrium state from the ‘no control’ scenario for the initial conditions.

## 3. Results

### 3.1. Dynamics of *T. gondii* transmission in cat and rodent populations

**Figure 2.**
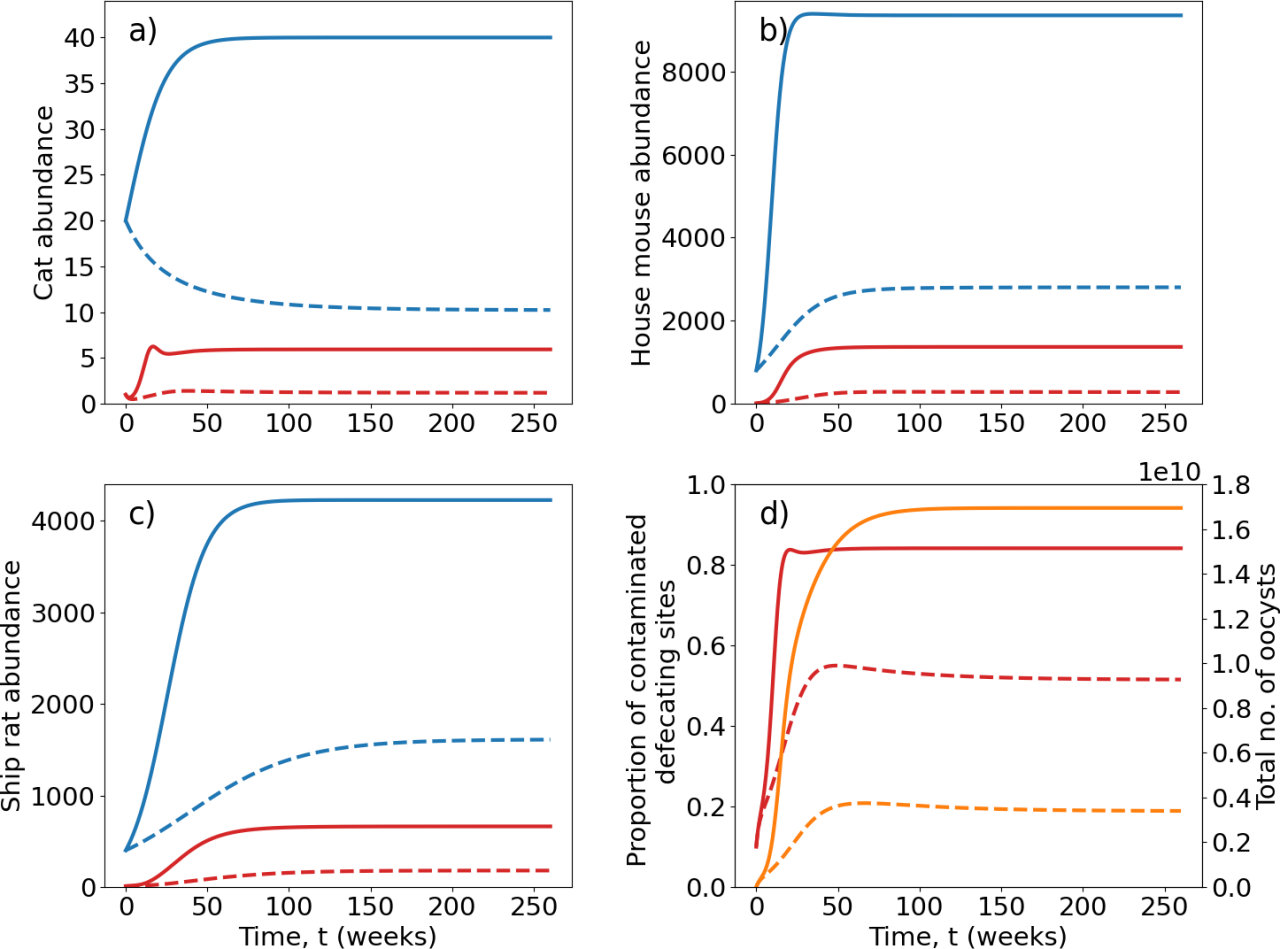
Population growth and transmission dynamics for (*a*) feral cats; (*b*) house mice; (*c*) ship rats; plus (*d*) environmental contamination, in the absence of control (solid lines) and for high cat and rodent control (sustained 75, 70 and 62% reduction in equilibrium population sizes of cats, house mice and ship rats) (dashed lines) in a hypothetical 10 km^2^ area of farmland. In (*a*–*c*) blue denotes total population size and red the number of infected individuals in cat and rodent populations over time. For (*d*) the proportion of contaminated defecation sites is shown in red and the total number of oocysts in the environment in orange over 260 weeks (5 years). Parameter values are as given in table 1, for a ‘high environmental contamination’ scenario.

**Figure 3.**
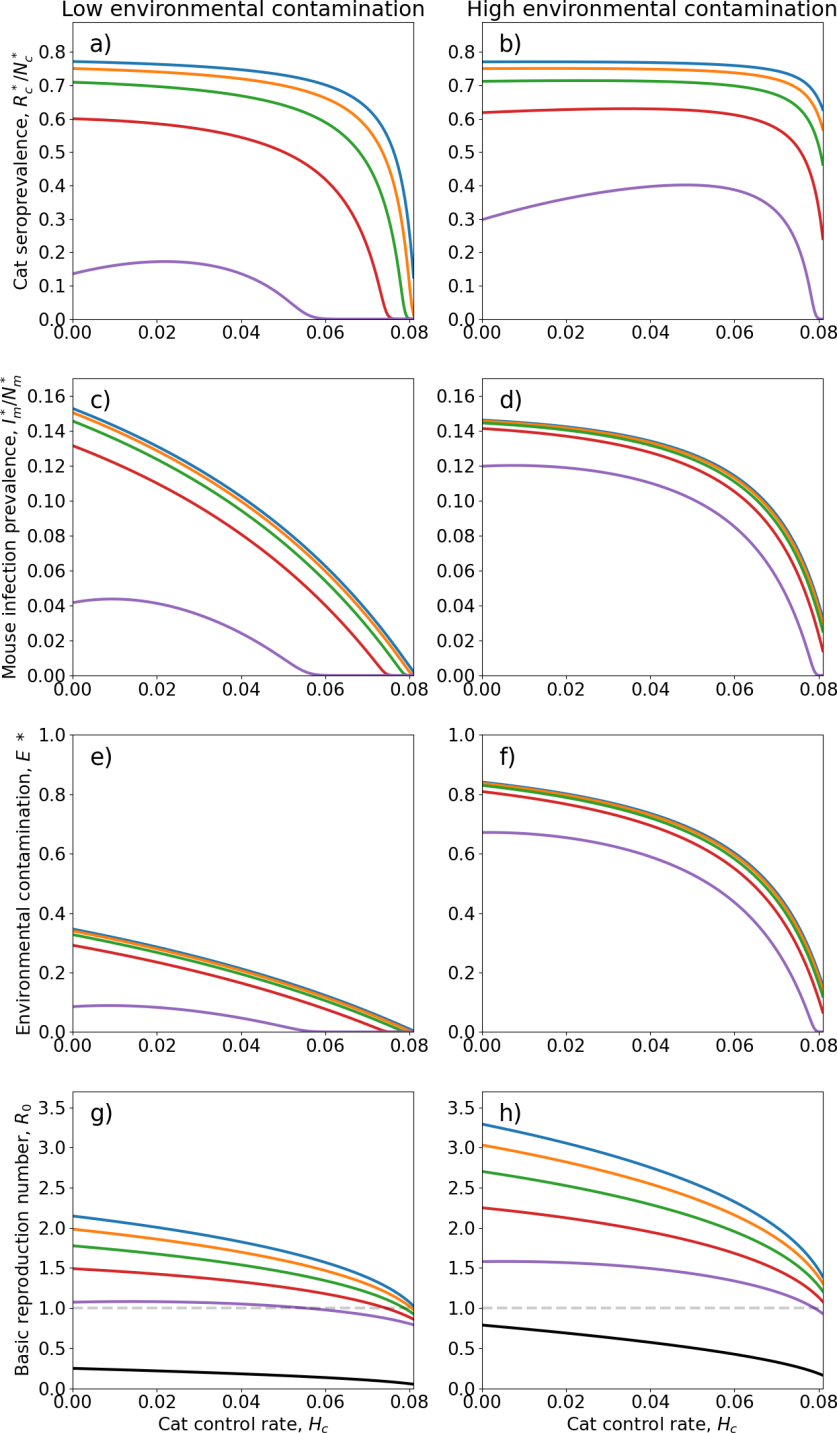
Effectiveness of varying rates of feral cat and rodent control for managing spread of *T. gondii* under two levels of environmental contamination. Changes in (*a,b*) cat seroprevalence; (*c,d*) house mouse infection prevalence; (*e,f*) environmental contamination; and (*g,h*) reproduction number $R_{0}$ at equilibrium, over varying cat control rate. (*a,c,e,g*) The ‘low’ environmental contamination scenario ( λ=0.1/16 , $\beta_{m}$ = 2.3/52, $\beta_{r}=1.05/52$ ). (*b,d,f,h*) The ‘high’ environmental contamination scenario ( λ= 1/16, $\beta_{m}$ = 0.9/52, $\beta_{r}=0.45/52$ ). Cat control rates of $H_{c}=$ 0.021, 0.042, 0.063 and 0.080 cat^−1^ week^−1^ correspond to 25, 50, 75 and 95% reductions in cat population size, respectively. Results shown for different scenarios of ‘no control’ (blue), ‘low’ (orange), ‘moderate’ (green), ‘high’ (red), ‘extreme’ (purple) control applied to both rodent species (see $H_{m}$ and $H_{r}$ values in table 1) and rodent elimination (black; equation (2.6)). The percentage reductions in house mouse and ship rat populations achieved by control depend on the control rates of all three species. The grey dashed line in (g,*h*) indicates $R_{0}=1$ threshold, below which *T. gondii* transmission is eliminated.

We first consider the dynamics of *T. gondii* spread in a farmland environment in the absence of feral cat or rodent control, $H_{c}=H_{m}=H_{r}=0$ (figure 2, solid lines). Solving the model using the parameter values in table 1 under the ‘high’ environmental contamination scenario, cat and rodent populations undergo logistic growth to equilibrium abundances of ${{{N_{c}}^{*}=K}_{c}}^{*}=40$ , $N_{m}^{*}=9351$ and $N_{r}^{*}=4227$ , corresponding to densities of 4 cats km^−2^, 935.1 house mice km^−2^ and 422.7 ship rats km^−2^ (figure 2a–c). Infection prevalences in rodent populations, determined by our choice of $\beta_{m}{,\beta }_{r}$ values, were $\frac{I_{m}^{*}}{N_{m}^{*}}=$ 0.15 in house mice and $\frac{I_{r}^{*}}{N_{r}^{*}}=$ 0.16 in ship rats at equilibrium, while the cat population reached an active infection prevalence of $\frac{I_{c}^{*}}{N_{c}^{*}}$ = 0.15 and seroprevalence of $\frac{R_{c}^{*}}{N_{c}^{*}}$ = 0.77. The model predicted a high proportion $E^{*}=$ 0.84 of defecation sites were contaminated and a total of $O^{*}=$ 1.69 × 10^10^ oocysts in the environment at equilibrium (figure 2d). Under the low environmental contamination scenario the model predicts similar population sizes, prevalences and number of oocysts but a lower proportion $E^{*}=$ 0.35 (figure 3).

Under a scenario of high cat and rodent control (i.e. one that achieves a sustained 75, 70 and 62% reduction in equilibrium population sizes of cats, house mice and ship rats, respectively), prevalences and environmental contamination are both reduced, but *T. gondii* infection persists (figure 2, dashed lines). In the following sections we systematically vary control rates to assess the effectiveness of cat and rodent control for reducing transmission and calculate the basic reproduction number $R_{0}$ for the system to explore the feasibility of eliminating *T. gondii*.

### 3.2. Environmental transmission cycle in cats (rodent elimination)

*T. gondii* will not persist in the environment or cat population if $R_{0}<1$ ; therefore from equation (2.6) it follows that in the absence of rodent prey, *T. gondii* can be eliminated for cat control rates that satisfy


(3.1) $$max\left(0,\;r_{c}-\frac{d_{0}\left(b_{c}+\gamma \right)r_{c}}{\beta_{c}K_{c}\lambda }\right)<H_{c}<r_{c}.$$

**Figure 4.**
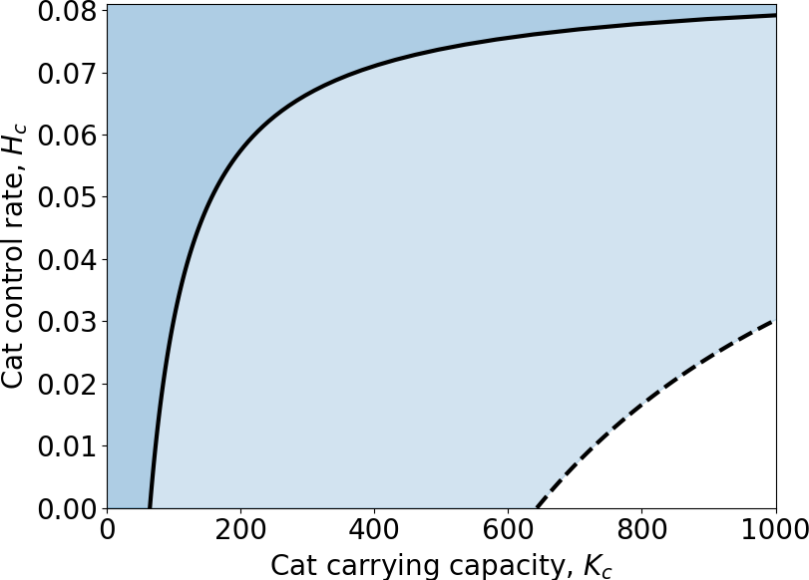
The $R_{0}=1$ threshold (dashed for low environmental contamination scenario; solid for high contamination) for sustained environmental transmission of *T. gondii* in the absence of rodent prey (i.e. rodent elimination), as a function of cat carrying capacity $K_{c}$ and cat control rate $H_{c}$ . The light- and dark-blue regions indicate values of $K_{c}$ and $H_{c}$ for which $R_{0}<1$ in the ‘low’ and ‘high’ environmental contamination scenarios, respectively.

For the default parameter values used here for a New Zealand farmland environment (table 1), $r_{c}-\frac{d_{0}\left(b_{c}+\gamma \right)r_{c}}{\beta_{c}K_{c}\lambda }=-1.2755$ under the low environmental contamination scenario and -0.0514 under high environmental contamination. This means elimination of *T. gondii* is achieved for any control rates $H_{c}\geq 0$ , i.e. if rodent populations are eliminated or naturally absent, *T. gondii* will not be able to spread by environmental transmission alone, even without controlling cats. However, $R_{0}$ also depends on cat carrying capacity $K_{c}$ (see equation (2.6)) and for sites supporting higher values of $K_{c}\;>\;\frac{d_{0}\left(b_{c}+\gamma \right)r_{c}}{\beta_{c}\lambda \left(r_{c}-H_{c}\right)}$ , the parasite can persist solely through the environmental transmission cycle. Therefore, for $K_{c}>643$ and $K_{c}>64$ under the low and high environmental contamination scenarios, respectively, cat control is required for *T. gondii* elimination (figure 4) even in areas where rodents have been eliminated. Though complete elimination of rodent prey is currently unrealistic in a rural farmland setting, this scenario is still useful to consider as it indicates that even extreme rodent control could fail to eliminate *T. gondii* transmission in areas with large cat populations.

### 3.3. Effectiveness of feral cat and rodent control for managing *T. gondii*

We compare the reductions in prevalence, environmental contamination and $R_{0}$ achieved by different intensities of cat and rodent control (figure 3). Results for house mouse and ship rat prevalence are very similar so we only present results for house mice. Environmental contamination at equilibrium $E^{*}$ and $R_{0}$ are sensitive to changes in the value of contamination rate λ (electronic supplementary material, figure S4). We therefore consider two scenarios representing ‘low’ and ‘high’ levels of environmental contamination. In the absence of feral cat and rodent control, the basic reproduction number derived from the full model equation (2.2) is $R_{0}=$ 2.1 and 3.3 for the low and high environmental contamination scenarios, respectively. Cat control, which directly removes $H_{c}I_{c}^{*}$ infected individuals per week, is more effective for reducing the total number of oocysts in the environment than rodent control, where changes in oocyst levels occur indirectly through reduced predation on infected rodents. However, the modelled transmission dynamics depend not on the number of oocysts but on the proportion of defecation sites that are contaminated $E^{*}$ . Since $E^{*}$ is relatively insensitive to changes in control rates (figure 3e,f), in general, very high levels of cat and rodent control are required to significantly reduce prevalence (figure 3a–d) and $R_{0}$ (figure 3g,h).

Increasing rodent control rates from ‘low’ to ‘high’ intensity has minimal impact, with most change in environmental contamination and prevalences achieved as cat control rates increase beyond high control (figure 3a–f). Rodent control alone can reduce transmission, with extreme rodent control achieving a 50% reduction in $R_{0}$ (compared to no control), and an 82% reduction in cat seroprevalence and total oocysts to $R_{0}=$ 1.1, $R_{c}^{*}/N_{c}^{*}$ = 0.14 and $O^{*}=$ 0.30 × 10^10^ respectively, in the low environmental contamination scenario. A smaller 61% reduction in cat seroprevalence and number of oocysts is achieved by extreme rodent control under high environmental contamination, to $R_{0}=1.6$ , $R_{c}^{*}/N_{c}^{*}$ = 0.30 and $O^{*}=$ 0.65 × 10^10^. With $H_{c}=0$ this is insufficient to eliminate *T. gondii* (i.e. when $R_{0}<1$ ) in either contamination level scenario. However, as discussed, if rodent control eliminates both rodent populations then *T. gondii* cannot spread through the environmental transmission cycle alone and the parasite is eliminated without the need for cat control (equation (2.6)).

**Figure 5.**
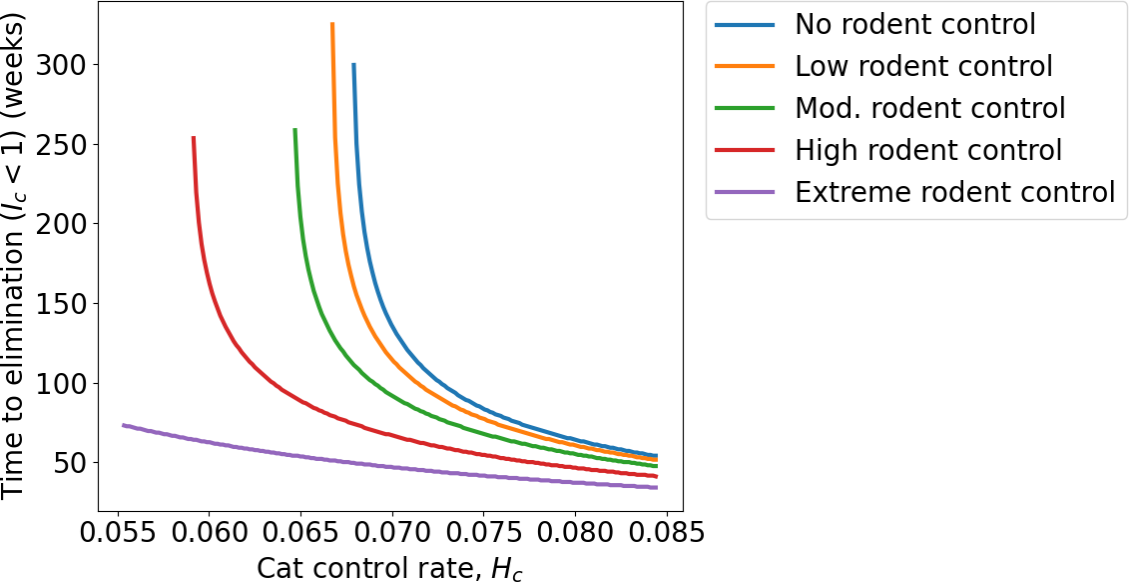
Time to elimination (weeks) of *T. gondii* under a scenario of low environmental contamination, λ=0.1/16 , $\beta_{m}$ = 2.3/52, $\beta_{r}=1.05/52$ , assuming the system is initially at its unmanaged equilibrium state, control begins at *t* = 0, and for varying rates of cat and rodent control.

Under low environmental contamination (figure 3a,c,e,g), *T. gondii* elimination is achievable: (i) for $H_{c}>0.0806$ (95% reduction from uncontrolled cat population size) with low rodent control (18% mouse and 7% rat reduction); (ii) for $H_{c}>0.0790$ (93% reduction from uncontrolled cat population size) with moderate rodent control (43% mouse and 33% rat reduction); (iii) for $H_{c}>0.0745$ (88% reduction) with high rodent control (69% mouse and 59% rat reduction); or (iv) for $H_{c}>0.0554$ (65% reduction) with extreme rodent control (90% mouse and 82% rat reduction) (figure 3g). In electronic supplementary material, D, we also show how $R_{0}$ varies as a continuous function of house mouse and ship rat control rates, for discrete levels of feral cat control (electronic supplementary material, figure S8). We assessed the period of sustained control required to eliminate *T. gondii* by assuming the system is initially at its unmanaged equilibrium state and control begins at *t* = 0. With $H_{c}$ at these threshold values and setting $I_{c}<1$ as the threshold for declaring *T. gondii* elimination, a minimum 59, 57, 56 or 73 weeks of sustained control is required to eliminate the parasite in the cat population under low, moderate, high and extreme rodent control, respectively (figure 5). Defining a stricter elimination threshold, $I_{c},\;\;I_{m},\;\;I_{r}<1$ , it takes decades to achieve elimination as the parasite persists in rodent populations through vertical transmission and abundance of infected rodents approaches zero asymptotically.

In the high environmental contamination scenario, prevalence (figure 3b,d) and environmental contamination (figure 3f) are relatively insensitive to increases in cat control rate up to a level of high cat control ( $H_{c}=0.063$ , i.e. 75% reduction in cat population size), with most impact on transmission achieved at control rates above this threshold (figure 3b,d,f,h). With rodent control at ‘high’ levels or less, *T. gondii* elimination is not achievable for $H_{c}\leq 0.080$ (i.e. cat control rates up to ‘extreme’ level) (figure 3h), though high rodent control combined with extreme cat control considerably reduces $R_{0}$ by 66%, cat seroprevalence by 57% and total oocysts by 98%, to $R_{0}=1.1$ , $R_{c}^{*}/N_{c}^{*}$ = 0.33 and $O^{*}=$ 0.04 × 10^10^, respectively. With extreme rodent control, *T. gondii* elimination is possible but only for cat control rates $H_{c}>0.079$ . Under extreme control of both cats and rodents (corresponding to 95%, 89% and 77% reductions in cat, mouse and rat population sizes, respectively), $R_{0}=0.97$ and a minimum 42 weeks of sustained control are required for elimination ( $I_{c}<1$ ). Unexpectedly, under extreme rodent control, cat seroprevalence increases with increasing cat control up to approximately $H_{c}$ = 0.06, then declines again for further increases in $H_{c}$ (figure 3b). Under extreme rodent control, infected mice increase slightly with $H_{c}$ up to approximately $H_{c}$ = 0.06, and infected rats increase with $H_{c}$ under all levels of rodent control.

## 4. Discussion

Despite the significant health impacts of *T. gondii*, the likely efficacy of potential management strategies for reducing its spread in rural environments is poorly understood. Our modelling of *T. gondii* transmission dynamics between feral cat, ship rat and house mouse populations, and the environment, highlights the challenge of attempting to reduce or eliminate transmission in New Zealand farmland through feral cat and rodent control alone. Our general conclusion is that very high levels of sustained feral cat and rodent control are necessary to substantially reduce environmental oocyst loads and prevalence in rural environments. Local elimination of *T. gondii* infection in cats may be possible under high levels of combined cat and rodent control. However, even if infection is successfully eliminated from cat populations, our model suggests the parasite could persist for long periods in rodent populations through vertical transmission to offspring. This means that infection to cats will resume once cat and rodent control are eased below the threshold control rates required for elimination. Immigration of infected cats into the management area, which is not accounted for in our closed-system model, will also hinder elimination attempts. Continuing cat control or introducing barriers to cat movement could extend the period of elimination. While we chose initial population densities representative of New Zealand farmland environments, real densities will vary over sites and seasonally and management of *T. gondii* may be more achievable for areas with smaller populations than those considered here.

Achieving such sustained (i.e. longer than one year), large reductions in rodent numbers will generally not be feasible for ship rats and mice on farmland unless large-scale management operations (e.g. landscape-level toxic baiting) are employed. Current control tools (traps and toxic baits) can achieve high levels of feral cat population reduction in production landscapes and natural areas (e.g. forests and drylands) [57] although the cost-effectiveness of deploying such methods for toxoplasmosis management is unknown. A major limitation on farmland, however, is the likely presence of domestic and stray cats in addition to truly feral animals. Management of free-ranging cats is a highly contentious issue in New Zealand [57] and it is unlikely that social licence exists in many rural areas at the present time for large-scale cat trapping or poisoning.

Greater control efforts are required for effective management of systems with high environmental contamination rates or high contact rates between rodents and *T. gondii*-contaminated environments. These rates are largely unknown for rural areas yet they have a strong influence on transmission dynamics, therefore obtaining measures of these parameters should be a priority for future field studies. While feral cat and rodent management may reduce environmental oocyst loads in rural areas, the extent to which this mitigates risk to livestock and wildlife will depend strongly on the susceptibility of different species to toxoplasmosis, the spatial distribution of contamination, and rates of diffusion for oocysts in soil and transportation in waterways.

Combining cat and rodent control with additional management methods that limit oocyst diffusion could improve the efficacy of management [6]. For example, wetlands can reduce transmission from land to sea by trapping oocysts in sediment and vegetation, so protection and restoration of wetland habitats may enhance oocyst retention [8]. Nevertheless, preventing transmission of oocysts into coastal environments is likely to be incredibly challenging given the widespread distribution of *T. gondii* and the additional oocyst loading from domestic cats into urban stormwater and wastewater systems. Although past models have indicated that cat vaccination could be an effective management method for reducing *T. gondii* transmission in theory [20], there are currently no commercially available cat vaccines and achieving high vaccination coverage seems infeasible for large feral cat populations.

Surprisingly, under extreme rodent control, cat seroprevalence and the abundance of infected rodents can increase slightly with cat control if insufficient cat control effort is applied. Extreme rodent control reduces populations to such low abundance that predation rates are small. Elasticity analyses by Lélu *et al*. [22] showed that for predation rates below 0.18 prey cat^−1^ week^−1^ the transmission cycle between cats and environment contributes more to the spread of *T. gondii* than does the predator–prey transmission cycle. Control of large cat populations could release small, highly suppressed rodent populations (particularly rats, for the parameters used here) from predation pressure, thereby driving increases in rodent population sizes, number of infected rodents, and transmission of *T. gondii* to the remaining cats via predation. Below a critical threshold rate of cat control, this rodent release due to cat control may counteract the reduction in transmission from controlling infected cats. This has implications for rural sites with low rodent densities and strong top-down effects of cat predation on rodents, where the environmental transmission cycle contributes more to spread of *T. gondii* than the predator prey cycle. In such cases, it may be counterproductive to control cats for toxoplasmosis management, unless cat control rates can be sustained at sufficiently high levels as to overcome this phenomenon.

The scarcity of studies reporting prevalence of *T. gondii* in New Zealand’s species and environments makes it difficult to assess which of our modelling scenarios are most realistic. A small New Zealand study found a seroprevalence of 61% in owned cats [6]. Prevalence of *T. gondii* is thought to be higher in feral cat populations compared to domestic cats and our model predicted a cat seroprevalence of 77% in the absence of control, which is broadly consistent with prevalences of 84 to 87% in feral and stray cat populations in Tasmania, Australia [58] and in western Amazon farmland, Brazil [59]. Studies of the spatial distribution of oocyst-contaminated soil in rural environments have reported proportions of contaminated soil samples ranging from 1.3% (swine farm, USA; cat seroprevalence 68.3%) [60] to 66.3% (highly contaminated areas around dairy farm buildings, France) [61]. The proportion $E^{*}=0.35$ , predicted under the scenario of low environmental contamination, lies in this range and is close to the 29.2% soil contamination observed by Gotteland *et al.* [62] in a rural area in France. The higher proportion $E^{*}=0.84$ , predicted under the high contamination scenario, may be more representative of urban or peri-urban environments. Adding to the uncertainty, there is considerable variation in the virulence and disease consequences of different types of *T. gondii* strains [63], yet most prevalence studies to date have not documented the strains being detected.

Our model has several limitations. For instance, it does not account for transmission of *T. gondii* through other intermediate hosts that are also prey to cats in New Zealand, such as lagomorphs and birds. In the Langham [12] cat diet study in Hawke’s Bay farmland, rabbits were scarce (approx. 0.05 rabbits ha^−1^) at the study site and house mice were the most frequently encountered remains in scats; while rats (mainly ship and Norway rats) were the most important component by weight. Other studies in New Zealand farmland habitat have found lagomorphs or birds are preferred prey [64,65]. Global reviews have reported *T. gondii* prevalence ranging from 1 to 38% in wild lagomorphs [66] and a pooled prevalence for wild birds of 18% [67]. It therefore seems reasonable to assume that rodents play the most significant role in transmission to cats at farmland sites with low lagomorph densities but cat and rodent control may be less effective in areas where lagomorphs or birds are more common prey. While we model deterministic logistic growth of cat and rodent populations, real rodent population dynamics are generally driven from the bottom up, with abundances fluctuating erratically between years in response to changes in food supply [68]. This between-year variation will affect the transmission dynamics and efficacy of control. For ecosystems with highly irruptive rodent dynamics, timing control operations to coincide with irruptions could be a more effective management strategy [69]. Our deterministic model is best suited for describing large, well-mixed populations. For small, spatially heterogeneous populations where random events and the spatial distribution of individuals have a strong influence on outcomes (such as the probability of elimination), a stochastic, spatially explicit model incorporating parameter uncertainty would be more appropriate (e.g. [25]).

We assume that cats only shed oocysts after initial infection with *T. gondii*; however, recent studies suggest repeat oocyst shedding in free-ranging felids may be more common than initially thought [70]. Our choice of decontamination rate assumes oocysts remain infective in the environment for an average of 100 days, which is in the range of oocyst survival times reported for soil at 15–30°C [35,71,72]. Other studies have reported longer survival times of up to 410 days in water [73] and up to 18 months in soil [71]. Including repeat shedding events in the model and/or reducing the decontamination rate would increase environmental oocyst load, leading to higher prevalence of *T. gondii* in rodent and cat populations, and reduced management efficacy.

Studies have shown a reciprocal relationship between house mouse and ship rat abundance in New Zealand forests, probably driven by biotic interactions (predation and/or competition of ship rats on mice). While we did not explicitly model these interactions, we chose carrying capacities that represent a farmland environment with higher densities of house mice than ship rats, in line with [50,51]. However, if these biotic interactions are important drivers of rodent population dynamics and if the management regime involves different levels of control effort for each rodent species it would be useful to include them explicitly in future applications of the model. If appropriate data were available, it would also be straightforward to include oocyst dose–response relationships for native wildlife or livestock species (or humans, following [23]) in the model, to quantify the reduction in risk of infection and fatality under different management regimes.

Our sensitivity analyses highlighted three parameters whose values are unknown for rural environments yet have a strong influence on transmission dynamics: (i and ii) environmental transmission rates in the two rodent species $\beta_{m}$ and $\beta_{r}$ ; and (iii) environmental contamination rate λ . These parameters will be challenging to measure directly but it may be possible to infer their values from measures of infection prevalence and the abundance and distribution of oocyst contamination in New Zealand’s rural environments. The measurement of these three factors is therefore a priority for future field studies. Evidence of vertical transmission rates in ship rats is highly limited, although our sensitivity analyses suggested that changes in rates of vertical transmission in rodents are important for probabilities $p_{m},p_{r}$ greater than 0.7, but less influential below this threshold. The value we used is towards the upper limit of the range of observed rates for house mice so if rates are lower for New Zealand rodent populations this will reduce the duration of control required to achieve elimination but is otherwise unlikely to strongly affect our findings.

In conclusion, our model of *T. gondii* transmission dynamics shows that trapping and/or toxic baiting of feral cat and rodent populations is a potentially viable approach for managing toxoplasmosis in rural environments, but only if very high levels of population control can be sustained. Such management seems likely to be feasible only in areas with small, isolated cat populations, low contamination levels and/or low contact rates between rodents and contaminated environment.

## Data Availability

This study did not use any datasets. All results were simulated using a mathematical model, with values for model parameters either drawn from literature or assumed; details of these parameter value sources are provided in table 1, Methods and electronic supplementary material A [74]. We provide detailed descriptions of the model formulation in Methods so that the model can be easily reproduced, and model code (programmed in Python) is available in the electronic supplementary material.
